# Supplementary material for: Relation of Alcohol Intake to Kidney Function and Mortality Observational, Population-Based, Cohort Study
Source: Nutrients. 2022 Mar 18;14(6):1297. doi: 10.3390/nu14061297 (PMC8954827; doi:10.3390/nu14061297)
Supplement: Supplementary file 1 [file nutrients-14-01297-s001.zip › nutrients-1624025-supplementary (1)-done.pdf]

nutrients

SUPPLEMENTARY MATERIALS

Supplementary Table S1 - Italian standards of volume, alcoholic graduation, alcohol equivalents of alcohol-containing servings.

Supplementary Table S2 – Simple correlation coefficient of covariates with alcohol intake in 2069 adult examinees with complete data at Exam-1, Exam-2, and Exam-3.

Supplementary Table S3 – Systolic pressure, diastolic pressure, and antihypertensive drug treatment by exam and stratum of alcohol intake in 2069 adult examinees with complete data at Exam-1, Exam-2, and Exam-3.

Supplementary Table S4 – Descriptive statistics at Exam-1 in examinees participating in all exams, in examinees dead during follow-up, and in examinees not dead and lost to follow-up.

Supplementary Table S5 – Cross-sectional analyses: multi-variable linear regression models for data of Exam-1 with eGFR regressed over stratum of alcohol intake and covariates in examinees that did not take part in Exam-2 and/or Exam-3.

Supplementary Table S6 – Cross-sectional analyses: multi-variable linear regression models with eGFR regressed over alcohol intake and covariates at Exam-1, Exam-2, and Exam-3.

Supplementary Table S7 – Longitudinal analyses: multi-variable linear regression models with annualized eGFR change and eGFR slope regressed over alcohol intake and covariates.

Supplementary Table S1 - Italian standards of volume, alcoholic graduation, alcohol equivalents of alcohol-containing servings.

Beverage	Volume	Alcoholic graduation	Alcohol equivalents per standard serving
Wine	125 mL	12%	11.8 g
Beer	333 mL	5%	13.1 g
Aperitif or cocktail	75 mL	20%	11.8 g
Spirits or liquors	40 mL	40%	12.6 g

Alcohol density = 0.789 g/mL

Società Italiana di Nutrizione Umana (SINU). Livelli di assunzione di riferimento di nutrienti (LARN) – IV Revisione. 2014 - ISBN 978 88 90685 22 4

Supplementary Table S2 – Simple correlation coefficient of covariates with alcohol intake in 2069 adult examinees with complete data at Exam-1, Exam-2, and Exam-3.

Covariates in analyses	Exam-1	Exam-2	Exam-3
Sex, men/women = 1/0	0.440***	0.500***	0.505***
Age, year	0.178***	0.113***	0.078***
Urinary sodium/creatinine ratio	-0.039^{ns}	-0.083*	not assessed
Urinary potassium/creatinine ratio	0.037	-0.058**	not assessed
Urinary urea nitrogen/creatinine ratio	not assessed	-0.173***	not assessed
Education, years	-0.103***	-0.081**	not assessed
Urinary creatinine	0.346***	0.405***	0.401***
Body mass index	0.109***	0.077***	0.074***
Systolic pressure	0.073***	0.089***	0.074***
Diastolic pressure	0.124**	0.125***	0.036 ^{ns}
On antihypertensive drug, yes/no = 1/0	0.005 ^{ns}	-0.005 ^{ns}	0.019 ^{ns}
Serum total cholesterol	0.212***	0.133***	0.043 ^{ns}
Smoker, yes/no = 1/0	0.176***	0.093**	0.044*
Diabetes, yes/no = 1/0	0.053*	0.027 ^{ns}	0.072***

^{ns} = not significant (P>0.05); * P < 0.05; ** P < 0.01; *** P ≤ 0.001

Supplementary Table S3. Mean systolic pressure, mean diastolic pressure, and prevalence of antihypertensive drug treatment by exam and stratum of alcohol intake in 2069 adult examinees with complete data at Exam-1, Exam-2, and Exam-3. .

		Alcohol intake g/d				P for trend
		0	1-24	25-48	>48	
**Exam-1**	Number of examinees	601	899	230	339	
	Systolic pressure, mm Hg	127	125	129	129	0.047
	Diastolic pressure, mm Hg	76	75	77	79	<0.001
	Antihypertensive drug, %	10.6%	8.0%	6.1%	9.7%	0.396
Exam-2	Number of examinees	663	565	454	387	
	Systolic pressure, mm Hg	123	124	126	128	<0.001
	Diastolic pressure, mm Hg	74	75	76	78	<0.001
	Antihypertensive drug, %	15.5%	14.9%	13.4%	15.0%	0.591

Exam-3	Number of examinees	927	472	401	269	
	Systolic pressure, mm Hg	133	134	134	137	0.002
	Diastolic pressure, mm Hg	77	77	76	79	0.258
	Antihypertensive drug, %	42.1%	42.8%	47.9%	45.0%	0.111

Supplementary Table S4 – Descriptive statistics at Exam-1 in examinees participating in all exams, in examinees dead during follow-up, and in examinees not dead and lost to follow-up: prevalence for categorical variables, mean for non-skewed variables and median for skewed variables.

		Participating in all exams	With missing Exam-2 and/or Exam-3	
			**Dead during follow-up**	Lost to follow-up
Number of examinees		2069	992	1463
Men, % (n)		42.8%	50.9%	43.5%
Age, years		43	67	46
eGFR, mL/min x 1.73 m²		91	73	89
Alcohol intake	Median, g/d	12	12	12
	% with no intake	29.0	24.9%	28.4%
	% with intake 1-24 g/d	43.5%	40.7%	48.7%
	% with intake 25-48 g/d	11.1	14.8%	11.8%
	% with intake > 48 g/d	16.4%	19.6%	11.1%
Urinary sodium/creatinine, mmol/g		107	146	107
Urinary potassium/creatinine, mmol/g		29	35	29
Urinary urea nitrogen/creatinine, g/g		not assessed	not assessed	not assessed
Education, year		7.6	5.0	7.9
Urinary creatinine, g/24-hour		1.26	1.16	1.24
Body mass index, kg/m²		26.1	27.5	26.0
Systolic pressure, mm Hg		127	150	134
Diastolic pressure, mm Hg		76	79	77
On antihypertensive drug, %		8.8%	28.1%	15.4%
Serum total cholesterol, mg/dL		206	219	208
Smoker, %		15.6%	25.4%	13.9%
Diabetes, %		1.1%	7.9%	3.7%

eGFR = estimated glomerular filtration rate

Supplementary Table S5 – Cross-sectional analyses: multi-variable linear regression models for data of Exam-1 with eGFR regressed over stratum of alcohol intake and covariates in examinees that did not take part in Exam-2 and/or Exam-3. Regression coefficient (B), 95% confidence interval (*italic)*, and P value.

		Dead during follow-up	Lost to follow-up
**Number of examinees**		992	1463
Habitual alcohol intake, g/d	0 (non-drinker)	0 (reference)	0 (reference)
	1 – 24	B = 3.051 *(0.86/5.25)* P = 0.006	B = 2.254 *(0.44/4.07)* P = 0.015
	25 – 48	B = 4.261 *(1.46/7.06)* P = 0.003	B = 2.615 *(-0.05/5.28)* P = 0.055
	> 48	B = 6.184 *(3.44/8.93)* P < 0.001	B = 4.747 *(1.79/7.71)* P = 0.002

eGFR = estimated glomerular filtration rate

Covariates included in the models = sex and data of Exam-1 for age, education, log-transformed urinary sodium/creatinine ratio, log-transformed urinary potassium/creatinine ratio, urinary creatinine, body mass index, systolic pressure, diastolic pressure, antihypertensive drug treatment, serum total cholesterol, smoking, diabetes.

Supplementary Table S6 – Cross-sectional analyses: multi-variable linear regression models with eGFR regressed over alcohol intake and covariates at Exam-1, Exam-2, and Exam-3. Standardized regression coefficient (beta), 95% confidence interval (*italic*), P value (asterisks), and adjusted R-squared (R^2).

		Dependent variable		
**Independent variables**		**Exam-1 eGFR**	**Exam-2 eGFR**	Exam-1 eGFR
Habitual alcohol intake, g/d	0 (non-drinker)	reference	reference	reference
	1 – 24	0.012 ns *(-0.03/0.05)*	-0.011 ns *(-0.05/0.02)*	-0.020 ns *(-0.05/0.01)*
	25 – 48	0.078*** *(0.04/0.12)*	0.044 * *(0.01/0.08)*	0.014 ns *(-0.02/0.04)*
	> 48	0.123 *** *(0.08/0.17)*	0.093 *** *(0.05/0.13)*	0.053 *** *(0.02/0.09)*
Sex, men/women = 1/0		0.372 *** (0.23/0.52)	0.371 *** *(0.25/0.50)*	0.384 *** *(0.28/0.49)*
Age, years		-0.666 *** *(-0.74/-0.60)*	-0.786 *** *(-0.85/-0.72)*	-0.812 *** *(-0.86/-0.76)*
Education, years		-0.041 ns *(-0.08/0.01)*	-0.087 *** *(-0.13/-0.05)*	not assessed
Urinary sodium/creatinine ratio, log mmol/g		0.029 ns *(-0.01/0.07)*	0.039 * *(0.01/0.08)*	not assessed
Urinary potassium/creatinine ratio, log mmol/g		-0.014 ns *(-0.05/0.03)*	0.030 ns *(-0.01/0.07)*	not assessed
Urinary urea nitrogen/creatinine ratio, log g/g		not assessed	0.131 *** *(0.10/0.17)*	not assessed
Urinary creatinine, g/d		-0.299 *** *-(0.46/-0.13)*	-0.152 * *(-0.30/-0.01)*	-0.213*** *(-0.34/-0.09)*
Body mass index, kg/m^2		0.109 ** *(0.03/0.19)*	0.098 ** *(0.03/0.17)*	0.082 ** *(0.02/0.14)*
Systolic pressure, mm Hg		0.031 ns *(-0.02/0.08)*	0.062 ** *(0.02/0.11)*	0.054 ** *(0.02/0.09)*
Diastolic pressure, mm Hg		-0.038 ns *(-0.09/0.01)*	-0.064 ** *(-0.11/-0.02)*	-0.043 * *(-0.08/-0.01)*
Antihypertensive drug treatment, yes/no=1/0		-0.26 ns *(-0.06/0.01)*	-0.036 * *(-0.07/-0.01)*	-0.056 *** *(-0.09/-0.03)*
Serum total cholesterol, mg/100 mL		-0.087 *** *(-0.13/-0.05)*	-0.063 *** *(-0.10/-0.03)*	-0.036 ** *(-0.06/-0.01)*
Smoking, yes/no=1/0		0.001 ns *(-0.04/0.04)*	-0.007 ns *(-0.04/0.02)*	-0.013 ns *(-0.004/0.01)*
Diabetes, yes/no=1/0		-0.010 ns *(-0.04/0.02)*	0.032 * *(0.01/0.06)*	0.031 * *(0.01/0.06)*
Adjusted R^2		0.384	0.533	0.625

eGFR = estimated glomerular filtration rate

ns = not significant (P>0.05); * P < 0.05; ** P < 0.01; *** P ≤ 0.001

Supplementary Table S7 – Longitudinal analyses: multi-variable linear regression models with annualized eGFR change and eGFR slope regressed over alcohol intake and covariates. Standardized regression coefficient (beta), 95% confidence interval (*italic*), P value (asterisks), and adjusted R-squared (R^2).

**Independent variables**		Dependent variable		
		eGFR change from Exam-1 to Exam-2	eGFR change from Exam-2 to Exam-3	eGFR slope from Exam-1 to Exam-3
		Model 1	Model 2	Model 3
**Habitual alcohol intake, g/d**	0 (non-drinker)	0 (reference)	0 (reference)	0 (reference)
	1 – 24	0.010 ns *(-0.02/0.04)*	-0.023 ns *(-0.06/0.02)*	-0.004 ns *(-0.04/0.03)*
	25 – 48	0.010 ns *(-0.02/0.04)*	-0.016 ns *(-0.06/0.02)*	0.019 ns *(-0.02/0.06)*
	> 48	0.065 *** *(0.03/0.10)*	0.050 * *(0.01/0.10)*	0.064 ** *(0.02/0.10)*
Sex, men/women = 1/0		0.248 *** *(0.13/0.37)*	0.325 *** *(0.18/0.47)*	0.346 *** *(0.23/0.46)*
Age, years		-0.553 *** *(-0.62/0.49)*	-0.781 *** *(-0.86/-0.70)*	-0.760 *** *(-0.82/-0.70)*
eGFR, mL/min x 1.73 m^2		-0.951 *** *(-0.99/-0.92)*	-0.772 *** *(-0.82/-0.72)*	-0.965 *** *(-1.00/-0.93)*
Education, years		-0.055 ** *(-0.09/-0.02)*	0.024 ns *(-0.02/0.07)*	0.019 ns *(-0.02/0.05)*
Urinary sodium/creatinine ratio, log mmol/g		0.008 ns *(-0.02/0.04)*	-0.064 ** *(-0.11/0.02)*	-0.037 * *(-0.08/-0.01)*
Urinary potassium/creatinine ratio, log mmol/g		0.046 ** *(0.01/0.08)*	0.049 * *(0.01/0.09)*	0.047 ** *(-0.01/0.08)*
Urinary urea nitrogen/creatinine ratio, log g/g		not assessed	0.042 * *(-0.02/0.14)*	-0.037 * *(-0.07/-0.01)*
Urinary creatinine, g/d		-0.091 ns *(-0.23/0.05)*	-0.178 * *(-0.35/-0.01)*	0.211 ** *(-0.35/-0.08)*
Body mass index, kg/m^2		0.087 ** *(0.02/0.15)*	0.062 ns *(-0.02/0.14)*	0.083 ** *(0.02/0.15)*
Systolic pressure, mm Hg		0.014 ns *(-0.03/0.05)*	0.002 ns *(-0.05/0.05)*	0.059 ** *(0.02/0.10)*
Diastolic pressure, mm Hg		0.001 ns *(-0.04/0.04)*	-0.027 ns *(-0.07/0.02)*	-0.039 * *(-0.08/0.01)*
Antihypertensive drug treatment, yes/no=1/0		-0.018 ns *(-0.05/0.01)*	-0.013 ns *(-0.05/0.02)*	-0.051 ** *(-0.09/-0.02)*
Serum total cholesterol, mg/100 mL		0.014 ns *(-0.02/0.05)*	0.015 ns *(-0.02/0.05)*	0.012 ns *(-0.02/0.04)*
Smoking, yes/no=1/0		-0.010 ns *(-0.04/0.02)*	-0.025 ns *(-0.06/0.01)*	-0.015 ns *(-0.04/0.01)*
Diabetes, yes/no=1/0		0.018 ns *(-0.01/0.05)*	-0.025 ns *(-0.06/0.01)*	0.016 ns *(-0.01/0.04)*
Adjusted R^2		0.586	0.389	0.605

ns = not significant (P>0.05); * $P < 0.05$; ** $P < 0.01$; *** $P \leq 0.001$

Covariates included in Model 1 = sex and data at Exam-1 of age, eGFR, education, log-transformed urinary sodium/creatinine ratio, log-transformed urinary potassium/creatinine ratio, urinary creatinine, body mass index, systolic pressure, diastolic pressure, antihypertensive drug treatment, serum total cholesterol, smoking, and diabetes.

Covariates included in Model 2 = sex and data at Exam-2 of age, eGFR, education, log-transformed urinary sodium/creatinine ratio, log-transformed urinary potassium/creatinine ratio, log-transformed urinary urea nitrogen/creatinine ratio, urinary creatinine, body mass index, systolic pressure, diastolic pressure, antihypertensive drug treatment, serum total cholesterol, smoking, and diabetes.
Covariates included in Model 3 = sex, age, eGFR, antihypertensive drug treatment, smoking, and diabetes at Exam-1, and means of data available from Exam-1 to Exam-3 for education, log-transformed urinary sodium/creatinine ratio (not measured at Exam-3), log-transformed urinary potassium/creatinine ratio (not measured at Exam-3), log-transformed urinary urea nitrogen/creatinine ratio (measured at Exam-2 only), urinary creatinine, body mass index, systolic pressure, diastolic pressure, serum total cholesterol.
